# The etiology and outcome of non-traumatic coma in critical care: a systematic review

**DOI:** 10.1186/s12871-015-0041-9

**Published:** 2015-04-29

**Authors:** Marlene WB Horsting, Mira D Franken, Jan Meulenbelt, Wilton A van Klei, Dylan W de Lange

**Affiliations:** 1 Division of Anaesthesiology, Intensive Care and Emergency Medicine, University Medical Centre Utrecht, mailstop Q04.2.313, PO Box 85500, 3508 GA Utrecht, Netherlands; 2 Department of Intensive Care Medicine and National Poison Information Center, University Medical Center Utrecht, Heidelberglaan 100, 3584 CX, Utrecht, Netherlands

**Keywords:** Non-traumatic coma, Etiology, Outcome, Mortality, Prognosis

## Abstract

**Background:**

Non-traumatic coma (NTC) is a serious condition requiring swift medical or surgical decision making upon arrival at the emergency department. Knowledge of the most frequent etiologies of NTC and associated mortality might improve the management of these patients. Here, we present the results of a systematic literature search on the etiologies and prognosis of NTC.

**Methods:**

Two reviewers independently performed a systematic literature search in the Pubmed, Embase and Cochrane databases with subsequent reference and citation checking. Inclusion criteria were retrospective or prospective observational studies on NTC, which reported on etiologies and prognostic information of patients admitted to the emergency department or intensive care unit.

**Results:**

Eventually, 14 studies with enough data on NTC, were selected for this systematic literature review. The most common causes of NTC were stroke (6-54%), post-anoxic coma (3-42%), poisoning (<1-39%) and metabolic causes (1-29%). NTC was also often caused by infections, especially in African studies affecting 10-51% of patients. The NTC mortality rate ranged from 25 to 87% and the mortality rate continued to increase long after the event had occurred. Also, 5-25% of patients remained moderately-severely disabled or in permanent vegetative state. The mortality was highest for stroke (60-95%) and post-anoxic coma (54-89%) and lowest for poisoning (0-39%) and epilepsy (0-10%).

**Conclusion:**

NTC represents a challenge to the emergency and the critical care physicians with an important mortality and moderate-severe disability rate. Even though, included studies were very heterogeneous, the most common causes of NTC are stroke, post anoxic, poisoning and various metabolic etiologies. The best outcome is achieved for patients with poisoning and epilepsy, while the worst outcome was seen in patients with stroke and post-anoxic coma. Adequate knowledge of the most common causes of NTC and prioritizing the causes by mortality ensures a swift and adequate work-up in diagnosis of NTC and may improve outcome.

**Electronic supplementary material:**

The online version of this article (doi:10.1186/s12871-015-0041-9) contains supplementary material, which is available to authorized users.

## Background

Coma is a serious condition requiring immediate medical decision making upon arrival at the emergency department or intensive care unit (ICU). As coma can originate from many different etiologies and is life threatening, it represents a challenge for emergency or critical care physicians [1]. Approximately 5% of the patients present to the emergency department with an altered mental state and 1% of the admissions at the emergency department is due to coma [2]. Coma also is a frequent cause for subsequent admission to the ICU. Although there is no consensus on the precise cut-off point to define coma, in general a GCS lower than 10 points is used [3]. Coma, without a history of a traumatic event, is an accompanying feature of many different conditions, such as severe sepsis, poisoning and hepatic encephalopathy. These conditions or the coma resulting from it, can be fatal if they are not detected or treated adequately [4].

Usually, as a first diagnostic step a differentiation is made between traumatic and non-traumatic coma (NTC). However, the determination of the cause of NTC is a challenge for the physician. In order to make adequate treatment decisions for patients suffering from NTC, it is vitally important to differentiate its etiology. An important first discrimination should be made between structural causes and non-structural causes of NTC by means of a computer tomography (CT) scan [5]. Structural coma can be due to cerebral infarction, intracranial hemorrhage, intracranial malignancy and central nervous system infection (e.g. encephalitis or abscess). Non-structural coma include coma as a result of poisoning, epilepsy, extracranial infections, circulatory shock, post-anoxic, cardiac arrest, respiratory failure, metabolic problems (such as hypoglycemia, ionic and acid–base disorders, hypothermia), hepatic encephalopathy and uremic encephalopathy [4].

Clearly, knowledge of the most frequent causes of NTC may improve the management of these comatose patients. In this systematic review, we aimed to quantify the causes of non-traumatic coma in an acute medical setting, both on the emergency department or ICU and the mortality associated with it.

## Methods

### Literature search

**Figure 1:**
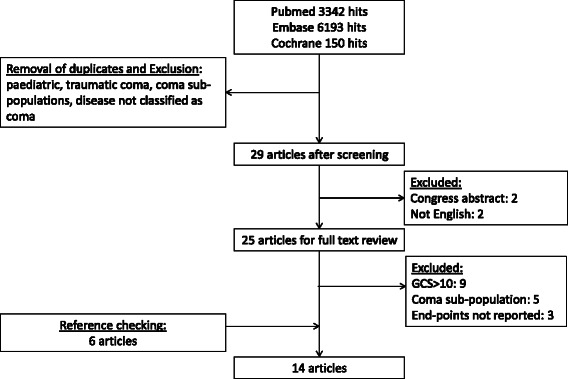
Systematic literature search.

A systematic literature search was performed in the Pubmed, Embase and Cochrane search engines (Figure 1). In the systematic search “altered mental state” or “non-traumatic coma” in the emergency department or intensive care unit was chosen as domain and the outcome was mortality. For each of these terms, keywords and medical subject heading (MESH) terms were defined to search in title and abstract. Altered mental state was included in the search strategy to prevent exclusion of articles reporting on non-traumatic coma with less strict terminology. The search entry for each database can be found as Additional file 1. All publications prior to May 16th, 2014 were included. All results were independently evaluated by two authors (MH and MF).

### Selection of studies

Only manuscripts written in English were selected. Inclusion criteria were retrospective or prospective observational studies on NTC. For the purpose of this review, NTC was defined as a GCS of 10 or less based on several studies included in this review [3,6-9]. Studies were selected if etiological data and/or prognostic data or mortality rates of patients presenting with NTC at the emergency department or ICU were provided. Exclusion criteria were pediatric patients, traumatic coma, NTC patient sub-populations, publications without prognostic, survival or mortality data of patients presenting with NTC. Search results were first evaluated on title and abstract only (Figure 1). Thereafter, a full publication screening was performed. References of eligible studies were reviewed to identify additional relevant publications. The Web of Science database was used to further identify relevant publications through citation indexing.

## Results

The literature search yielded 9,685 studies, which were evaluated according to the algorithm depicted in Figure 1. Twenty nine articles were selected after screening on title and abstract. Two search results were merely congress abstracts and two articles were not written in English. Twenty five articles were selected for full text review. Nine articles were subsequently excluded as the study only reported on patients with altered mental state (GCS < 15) and not exclusively on patients with GCS 10 or less, three articles were excluded because no information on mortality and NTC etiologies was provided, and five articles only reported on a specific coma subgroup. References of eligible studies were reviewed for additional relevant studies, which revealed an additional six publications. In total, 14 publications were selected for this review (Table 1).

**Table 1:** **Characteristics of included prospective observational studies**

Study	N	Setting	Inclusion	Exclusion	Mortality	Follow-up duration	Country
Esquevin 2013 [6]	65	University ED	GCS ≤ 10, ≥18 yrs,	Trauma, diagnosis such as meningitis, status epilepticus or drug abuse prior to NCCT and CTA, >24 hrs between event and NCCT and CTA, contraindication to iodinated contrast injection, increased or unknown creatinine level	52% after 3 months	3 months	France
Forsberg 2012 [21]†	865	University, non-surgical ED	GCS ≤10 maintained for ≥30 min., ≥18 yrs	Coma of unknown cause at discharge or psychogenic, unknown identity of the patient	27%	2 years	Sweden
Forsberg 2012 [9]†	875	University, non-surgical ED	GCS ≤10 maintained for ≥30 min., ≥18 yrs	Coma of unknown cause at discharge or psychogenic, >1 plausible coma etiology	26%	NS	Sweden
Weiss 2012 [18]	2189	University ICU	GCS < 8 in the first 24 hrs of ICU admission		48%	Until discharge	France
Forsberg 2009 [7]†	938	University, Non-surgical ED	GCS ≤10 maintained for ≥30 min., ≥18 yrs	NS	25%	Until discharge	Sweden
Greer 2012 [14]	500	University, ED, ICU, cardiac or neuroscience ICU	GCS <8	Coma because of sedating medications or traumatic coma	87% after 6 months	6-months	USA
Hamel 1995 [19]	596	University hospital or ICU	GCS ≤ 9 for ≥6 hrs	Trauma, drug intoxication, hypothermia, operative complication, diabetic ketotic coma, nonketotic hyperosmolar coma, thyrotoxicosis, myxoedema coma, hepatic encephalopathy, uraemia, hypo- or hypernatremia, hypo- or hypercalcaemia. Hospital discharge, death, brain death within 48 hrs of study eligibility	69% after 2 months	6-months	USA
Sacco 1990 [16]	169	University ICU	GCS ≤ 8, >10 yrs	Trauma, acute encephalopathy of unknown cause, coma existing > 72 hrs	54% after 2 weeks	2-weeks	USA
Levy 1981 [15]	500	University hospital	Coma at admission or during hospitalization, ≥12 yrs	Trauma, drugs	64% after 1 week	1-year	USA/UK
Tokuda 2003 [17]	115	Urban teaching hospital ED	GCS < 8, ≥15 yrs	Out of hospital cardiac arrest	NS	Until discharge	Japan
Owolabi 2013 [12]	194	University, non-surgical ED	GCS ≤ 8, ≥18 yrs	Trauma, post anaesthesia coma, coma due to sedative drugs or alcohol	49% after 1 month	1 month	Nigeria
Obiako 2011 [10]	200	University, ED	GCS ≤ 8, ≥16 yrs	Trauma, inconclusive diagnosis	NS	28 days	Nigeria
Sinclair 1989 [13]	139‡	University, ICU	GCS NS, ≥18 yrs	Trauma	NS	Until discharge	Zambia
Matuja 1987 [11]	150	University hospital	GCS ≤ 8 for ≥6 hrs	Transient unresponsiveness, impending death, postictal state, hypoglycaemia	61% after 1 month	1-month	Tanzania

CTA = computed tomographic angiography, ED = emergency department, GCS = Glasgow Coma Scale [7], ICU = intensive care unit, NCCT = noncontrast computed tomography, NS = not specified, POS = prospective observational study, RS = retrospective study.

† All publications by Forsberg et al. reflect data of the same patient population included between February 2003 and May 2005.

‡ The original publication included 170 patients, however 139 of 153 patients of which GCS was documented were selected for this analysis as only data of GCS ≤ 10 were included.

### Non-traumatic coma etiology

The most common causes for NTC were stroke (range: 6-54%), post-anoxic coma (range: 3-42%), poisoning (range: <1-39%) and metabolic (range: 1-29%) (Table 2). The prevalence of different causes varied greatly between studies. Infections, including central nervous system infections, were more prevalent in the African studies compared to the other included studies, 10-51% and 2-9% respectively [10-13]. Additionally, Owolabi et al. reported HIV related coma in 4% of patients in their cohort and Matuja et al. reported a high prevalence of cerebral malaria (40%), while none of the other studies reported on these etiologies as cause of coma [11,12].

**Table 2:** **Prevalence (in percentages) of non-traumatic coma etiologies**

Study reference		Esquevin [6]	Forsberg [9]	Weiss [18]	Greer [14]	Hamel† [19]	Sacco [16]	Levy [15]	Tokuda [17]	Owolabi [12]	Obiako [10]	Sinclair [13]	Matuja [11]
Structural	Stroke^1^	54	24	6	49	51	36^5^	37	29	24	33	22	13
	CNS infection	2	2	3	-	6	-	-	-	14	10	19	51
	Malignancy	3	2	-	-	3	-	-	<1	2	3	1	-
	Other neurological causes	-	-	1	-	-	7	-	11	-	-	1	-
Metabolic/Non-structural	Poisoning	-	39	19	-	<1	-	-	32	-	1	15	4
	Epilepsy	5	13	7	-	-	-	-	2	4	-	-	-
	Post-anoxic coma	3	7	23	40	31	36	42	-	-	4	-	-
	Respiratory	-	4	8	-	-	-	-	5	-	-	-	-
	Infection	-	4	-	-	9	-	-	4	19	11	-	-
	Metabolic^2^	31^4^	4	14	-	1	22	-	8	21	29	6	5
	Hepatic encephalopathy	-	<1	-	2	-	-	10	2	8	6	1	20
	Shock	-	-	21	-	<1	-	-	-	-	-	-	-
	Unclassified^3^	-	-	<1	-	-	-	-	2	8	-	22	-
	Miscellaneous	3	-	1	8	14	-	12	<1	-	-	-	8
	Eclampsia	-	-	-	-	-	-	-	-	-	-	13	-

^1^Stroke includes cerebral infarction, intracerebral hemorrhage and subarachnoid hemorrhage.

^2^The term metabolic includes endocrine, ionic and acid–base disorders and metabolic brain dysfunction (uremic encephalopathy).

^3^Unclassified means that it was not possible to label the event with a diagnosis.

^4^Includes poisoning, metabolic or immune disorders and respiratory failure.

^5^Percentage reflects focal (brain tumour, abscess, infarct, intraparenchymal hemorrhage) and generalized cerebral (meningitis, hydrocephalus, intraventricular hemorrhage, subarachnoid hemorrhage) events.

† More than one coma etiology could be scored in the study, resulting in a total percentage > 100%.

Reported prevalence of structural coma varied between 28-64% and non-structural coma between 37-75% of patients presenting with non-traumatic coma [6,9,11,14-17]. Of note, Weiss et al. only reported a structural coma prevalence of 9% (cerebrovascular accidents and infections of the central nervous system) [18]. It should however be noted that as this hospital does not have a neurological or neurosurgical ward, this is likely to influence the incidence of reported coma. Hamel et al. only reported a metabolic cause of coma in 1% of the patients and poisoning in less than 1% of the patients. The study excluded NTC as a result of drug intoxications and multiple causes of metabolic coma (Table 1) [19].

### Outcome

The primary outcome of interest for this review was mortality. The average mortality rate in NTC patients reported was 25-87% and varied markedly between studies (Table 1). Patients with stroke had the highest mortality (60-95%), followed by patients with post-anoxic coma (54-89%) (Table 3). In contrast, the lowest mortality was found for poisoning and epilepsy: 0-7% and 0-10%, respectively and 35-67% for poisoning in the African studies [8,10-13,16,18].

**Table 3:** **Mortality rates (in percentages) of non-traumatic coma etiologies**

Study reference	Esquevin [6]	Forsberg [21]	Weiss [18]	Greer [14]	Sacco [16]	Levy [15]	Owolabi [12]	Sinclair [13]	Matuja [11]
Stroke	83	60	68	88	-	74	69	93	95
CNS infection	0	-	17	-	-	-	-	38	39
Poisoning	-	2	7	-	0	-	-	35	67
Epilepsy	0	<1	10	-	-	-	-	-	-
Post-anoxic coma	50^2^	72	80	89	54	58	-	-	-
Respiratory	-	61	36	-	-	-	-	-	-
Infection	-	27	-	-	-	-	-	-	-
Metabolic^1^	15	16	20	-	48	-	-	86	43
Hepatic encephalopathy	-	-	-	80	-	49	-	-	90
Shock	-	-	80	-	-	-	-	-	-
Eclampsia	-	-	-	-	-	-	-	33	-
Unclassified	-	-	-	-	-	-	-	93	100

^1^The term metabolic includes endocrine, ionic and acid–base disorders and metabolic brain dysfunction.

^2^Includes poisoning, metabolic or immune disorders and respiratory failure.

Coma etiology seemed to influence outcome. Sacco et al. reported that coma as a result of poisoning had an eight times higher probability of awaking compared to coma of other etiologies [16]. Additionally, Forsberg et al. reported that coma as a result of poisoning not only resulted in a higher chance of awaking, but also in less neurological sequelae measured with the Glasgow-Pittsburgh scale [7,20]. In their cohort (n = 352), 2% of patients with coma due to poisoning experienced neurological sequelae defined as permanent vegetative state to moderated disability (disabled but independent) compared to 18% of patients with coma due to other non-traumatic causes [7]. However, Matuja et al. reported that 4 out of 6 patients with poisoning included in their study died and one patient remained in a permanent vegetative state [11].

Furthermore, Forsberg et al. reported that mortality increased from 27% at hospital discharge to 39% in the first year thereafter [21]. This mortality rate was much higher compared to the age matched 1-year mortality in the general population and this was seen in all coma etiologies. Also, even though poisoning and epilepsy had a low mortality rate during first admission, 2.4% and 0.9% respectively, the mortality rate raised to 11% and 16% after 1-year [21].

In addition to the reported mortality rates, permanent vegetative state or severe disability was an outcome measure in 3 of the 14 studies. For instance, Levy et al. reported a mortality of 61% of patients, while 12% of patients remained in permanent vegetative state and 11% of patients were severely disabled (defined as acceptable cognition but dependent on others for daily support) after 1 year [15]. Hamel et al. reported death in 69% of patients and severe disability (unable to perform ≥4 activities of daily living) in 20% of patients after 2 months [19]. Finally, Greer et al. reported 87% death and 5% moderate to severe disability as assessed by modified Rankin Scale [14].

Patients with a GCS of 3–5 had a significantly higher probability of death compared to patients with a GCS of 6–8 [12,16]. Forsberg et al. presented similar results. Patients with GCS 3–6 had a significantly higher mortality rate compared to a GCS of 7–10 [21].

## Discussion

We performed a systematic literature review on the etiology and outcome of non-traumatic coma (NTC) over the years 1981–2013. There was only a limited number of publications available on this subject (n = 14). The most frequently reported reasons for NTC were stroke, post-anoxic coma, poisoning and metabolic events. Infection appeared to be an important cause of NTC in African countries. Coma etiology and follow-up period influenced the reported NTC mortality rates, which ranged from 25% to 87%.

### Coma etiology

The most important reasons for NTC appeared to be: stroke, post-anoxic coma, poisoning and metabolic events. However, these categories are rather broad themselves. For example stroke can be divided into ischemic stroke and intracranial hemorrhage. Ischemic stroke has a lower mortality rate than intracerebral hemorrhage. In a large patient cohort (n = 31,629) the 30 day mortality was 11% for ischemic stroke and 37% for intracerebral hemorrhage [22]. Most included studies in our review lack sufficient detail to allow for a further subdivision of stroke. Nevertheless, it is important to realize that in contrast to hemorrhagic stroke, ischemic stroke only causes coma if the brainstem is involved or if there is a malignant MCA stroke.

Post-anoxic coma was identified as a second important cause of NTC. Generally, post-anoxic coma is the result of an out-of-hospital cardiac arrest. The mortality associated with post-anoxic coma ranged from 54% to 89%. Some of these differences might be explained by improvements in the care of post-anoxic patients through the years. The introduction of mild therapeutic hypothermia and the improvement of critical care have led to a decline of the hospital mortality of such patients [23]. Again, differences in severity of illness between the cohorts might explain the remainder of the differences in observed mortality.

Poisoning is another major reason for NTC. In the included studies the prevalence of intoxications ranged from <1% to 39%. The associated in-hospital mortality was low (2.4%) but the one-year mortality was 10.9%. In a recent large observational study of intoxicated patients admitted to the ICU Brandenburg et al. showed similar hospital mortality rates (2.1%) but a somewhat lower mortality during follow-up (their 2-year mortality was 9.3%) [24].

The in-hospital mortality associated with NTC as a result of metabolic derangements ranged from 15 to 86%. The group of metabolic NTC is a very diverse group but diabetic derangements and hepatic failure are the most prominent causes of metabolic NTC. Clearly, the cause of metabolic NTC is related to outcome. Diabetic ketoacidosis with associated coma is associated with mortality rates <1% while hyperglycemic hyperosmolar state is associated with mortality rates of 5-20% [25]. Hepatic encephalopathy has an even worse prognosis. Fichet et al. included 71 patients with severe hepatic encephalopathy admitted to the intensive care unit (ICU) of which 76% of patients were admitted with coma (the mean GCS at admission to the ICU was 7.7 ± 4). During the ICU stay, 35% of patients died and the 1 year-mortality was 54% [26].

### Limitations

We tried to include observational cohorts who used similar inclusion criteria for patients with NTC, being a GCS <10 and were able to include over 5,000 patients in our systematic review. Of note, Weiss et al. included an important number of patients in their report (N = 2189), which was by far the largest study included in this review [18]. Despite its size, our study has major drawbacks as we found a large variability in the different cohorts and outcomes. This is due to the differences in hospital settings (academic versus urban and the differences in physical setting within the hospital), differences in location (Western world versus Africa) and differences in in- and exclusion criteria for causes of NTC. The latter is illustrated by the exclusion of patients with an intoxication as cause of NTC in some studies, while in other intoxications appeared to be a major contributor to NTC [6,9,12,14,15,17-19]. This explains the very low prevalence of drug intoxication and metabolic causes of coma in the SUPPORT cohort of Hamel et al. Even so, within studies including intoxication as cause of NTC, there was a large difference in poisoned patients as a cause of NTC. For instance, a prevalence as high as 38% of patients was reported by Forsberg et al., while 19% was reported by Weiss et al. This might be due to the difference in coma severity, GCS ≤ 10 versus GCS < 8 as inclusion criterion in these studies, in addition to the in- and exclusion of specific etiologies in these studies [7,18]. Other causes of bias, based on in- and exclusion criteria, are that some studies allowed the inclusion of traumatic causes of coma and some studies also allowed for the coma to arise whilst already hospitalized [15,17,18]. Differences in hospital settings are also an important limitation in this analysis. Some hospitals, especially when they are specialized in certain patients’ categories, attract more severely ill patients or subgroups of patients. These differences will undoubtedly be reflected in the composition of the study population as well as their outcomes. In addition, it is important to identify the difference in organization structure and length of stay on the emergency department before the patient is admitted to the ICU. If patients only stayed a few hours in the emergency department and are subsequently transferred to the ICU, the outcome of the emergency department will be different from another emergency department that treats such patients for several days on the ED. Such organizational differences are very important to consider but very few studies provide these details. The relationship between NTC etiology and outcome might be further obscured if studies allow hospitalized patients to be admitted to the ICU or the ED. Again, such details are often lacking. We have included all patients with NTC who were admitted to the ED and/or ICU irrespective of the time spent on the ED.

Another major limitation is that some studies were over 25 years old. These results may no longer reflect current outcome as the care of critically ill patients has improved significantly over the last decades.

Finally, some publications unfortunately appeared to have studied the same population. For example, Forsberg et al. had three publications based on largely the same patient dataset. These three studies contained different etiological and prognostic data and were all included in our review [7,9,21].

## Conclusions

We have shown that the currently studied cohorts hamper thorough conclusion making on the NTC etiology and mortality as the patient cohorts were too heterogeneous. Despite these limitations, we advise to focus the diagnostic work-up in patients with NTC on: (1) stroke, (2) post-anoxic coma, (3) poisoning and (4) metabolic events, because these were the most important causes of NTC.

Clearly, a swift and adequate work-up in the diagnosis of NTC on the emergency department and ICU might improve outcome of such patients. Ideally, the diagnostic work-up aims to first identify those causes of NTC, where time is of essence and patients have the highest chances of clinical improvement if the cause of NTC is identified quickly. We therefore advocate that more standardized patient cohort studies are necessary to be able to confirm which etiologies are the most prominent in NTC and using solid endpoints to register morbidity and mortality in a standardized manner. These studies should include all patients presenting with NTC at the hospital, irrespective of the cause of NTC, and fixed outcome end-points should be used. Only so, will future opportunities for the improvement of patient outcome be identified in patients with NTC.

## Additional file

Additional file 1:
**Database search entries in Pubmed, Embase and Cochrane search engines.**

## Abbreviations

CNS: Central nervous system
CTA: Computed tomographic angiography
ED: Emergency department
GCS: Glasgow Coma Scale
HIV: Human immunodeficiency virus
ICU: Intensive care unit
NCCT: Noncontrast computed tomography
NS: not specified
NTC: non-traumatic coma
POS: prospective observational study
RS: retrospective study
MESH: Medial Subject Headings
UK: United Kingdom
USA: United States of America
