# Supplementary material for: The etiology and outcome of non-traumatic coma in critical care: a systematic review
Source: BMC Anesthesiol. 2015 Apr 29;15:65. doi: 10.1186/s12871-015-0041-9 (PMC4424591; doi:10.1186/s12871-015-0041-9)
Supplement: Additional file 1: — Database search entries in Pubmed, Embase and Cochrane search engines. [file 12871_2015_41_MOESM1_ESM.pdf]

Database search entries

The following search query was used in Pubmed: (intensive care [tiab] OR intensive care [MESH] OR intensive care unit* [tiab] OR intensive care units [MESH] OR emergency service [MESH] OR emergency department* [tiab] OR emergency service* [tiab] OR emergency unit* [tiab] OR ED [tiab]) AND (coma [tiab] OR coma [MESH] OR coma/etiology[MESH] OR coma/mortality* [MESH] OR coma/physiopathology* [MESH] OR coma/epidemiology* [MESH] OR coma/therapy [MESH] OR nontraumatic coma [tiab] OR non-traumatic coma [tiab] OR comatose [tiab] OR unconsciousness [MESH] OR unconsciousness [tiab] OR loss of consciousness [tiab] OR decreased consciousness [tiab] OR Glasgow Coma Scale* [tiab] OR Glasgow Coma Scale* [MESH] or decreased Glasgow Coma Score [tiab] OR altered mental status [tiab] OR altered mental state* [tiab]) AND (prognosis [tiab] OR prognosis [MESH] OR survival [tiab] OR survival [MESH] OR mortality [tiab] OR mortality [MESH] OR disease progression [tiab] OR disease progression [MESH])

The following search query was entered in Embase: ('prognosis'/syn or 'prognosis'/exp or 'prognosis' or 'survival'/syn or 'survival'/exp or 'survival' or 'mortality'/syn or 'mortality'/exp or 'mortality' or ('disease'/exp or disease and progression) and [humans]/lim) and (intensive and care or (intensive and care and unit*) or ('emergency' or 'emergency'/exp or emergency and service) or ('emergency' or 'emergency'/exp or emergency and department*) or ('emergency' or 'emergency'/exp or emergency and service*) or ('emergency' or 'emergency'/exp or emergency and unit*) or ed and [humans]/lim) and ('coma' or 'coma'/exp or coma or (nontraumatic and ('coma' or 'coma'/exp or coma)) or ('non traumatic' and ('coma' or 'coma'/exp or coma)) or comatose or 'unconsciousness' or 'unconsciousness'/exp or unconsciousness or (loss and of and ('consciousness' or 'consciousness'/exp or consciousness)) or (decreased and ('consciousness' or 'consciousness'/exp or consciousness)) or (glasgow and ('coma' or 'coma'/exp or coma) and scale*) or (decreased and glasgow and ('coma' or 'coma'/exp or coma) and score) or (altered and mental and status) or (altered and mental and state*) and [humans]/lim)

The following search query was used in the Cochrane library: 'intensive care OR intensive care unit* OR emergency service OR emergency department* OR emergency service* OR emergency unit* OR ED in Title, Abstract, Keywords and coma OR nontraumatic coma OR non-traumatic coma OR comatose OR unconsciousness OR loss of consciousness OR decreased consciousness OR Glasgow Coma Scale* OR decreased Glasgow Coma Score OR altered mental status OR altered mental state* in Title, Abstract, Keywords and prognosis OR survival OR mortality OR disease progression OR disease progression in Title, Abstract, Keywords. Reviews, other reviews and studies were searched in the Cochrane database.
